# Rituximab Monotherapy versus Rituximab and Bortezomib Combination Therapy for Treatment of Non-paraneoplastic Autoimmune Retinopathy

**DOI:** 10.18502/jovr.v17i4.12304

**Published:** 2022-11-29

**Authors:** Arash Maleki, Amanda Colombo, Sydney Look-Why, BA; Peter Y Chang, Stephen D Anesi, Stephen D Anesi

**Affiliations:** ^1^Massachusetts Eye Research and Surgery Institution, Waltham, MA, USA; ^2^Ocular Immunology and Uveitis Foundation, Waltham, MA, USA; ^3^Harvard Medical School, Boston, MA, USA; ^5^https://orcid.org/0000-0001-4760-8390; ^6^https://orcid.org/0000-0003-4760-8390

**Keywords:** Autoimmune Retinopathy, Bortezomib, Electroretinography, Non-paraneoplastic AIR, Rituximab, Visual Field

## Abstract

**Purpose:**

To study whether rituximab and bortezomib combination therapy is more effective than rituximab monotherapy in the treatment of non-paraneoplastic autoimmune retinopathy (npAIR).

**Methods:**

Retrospective case series involving six patients with npAIR, taking either rituximab and bortezomib combination therapy (three cases) or rituximab monotherapy (one case and two historical patients).

**Results:**

Patients on both treatment regimens showed stability in most of the visual function parameters during the one year of follow-up. Combination therapy resulted in improvement of scotopic combined rod and cone a-wave and b-wave amplitudes in all eyes where they were available (four eyes); however, rituximab monotherapy resulted in only two eyes with stable scotopic combined rod and cone a-wave and b-wave amplitudes, while four eyes showed a decrease in both a- and b-wave amplitudes. The average improvement in b-wave amplitude (50.7% ± 29.4% [range, 25–90%]) was higher than the average improvement in a-wave amplitude (35.7% ± 9.74 [range, 25–63%]). No severe adverse effects were reported.

**Conclusion:**

Rituximab and bortezomib combination therapy may not be more effective than rituximab monotherapy in npAIR patients for most of the visual function parameters; however, this combination therapy may be more effective in improving scotopic combined rod and cone a- and b-wave amplitudes. This may indicate the higher efficacy of combination therapy when there is involvement of the inner retina.

##  INTRODUCTION

Non-paraneoplastic autoimmune retinopathy (npAIR) was first reported in 1997.^[1]^ It is usually diagnosed in younger patients and found more frequently in females (63–66%).^[2,3]^ Patients with npAIR typically have a strong family history of systemic autoimmune diseases. While patients with paraneoplastic AIR usually develop a more rapid onset of symptoms and have more obvious electroretinographic changes, patients with npAIR have symptoms that often present sub-acutely and involve more subtle ERG changes.^[4]^ Clinical presentation is variable and likely dependent on the retinal cells targeted by the ARAs.^[5]^

Many different immunosuppressive medications have been studied for the treatment of autoimmune retinopathy (AIR); however, to date, there is no consensus on recommendations for specific treatment protocols due to the rarity of the condition and the difficulty in conducting randomized controlled trials.^[6]^

Rituximab is a monoclonal antibody that targets CD20+ B cells, which causes a reduction in systemic antibody levels and is a logical contender for the standard of treatment for AIR. Our previous study documented stabilization and/or improvement with the use of both rituximab as monotherapy and in combination with cyclophosphamide or bortezomib.^[7]^ Similarly, a number of individual case reports have been published describing varying levels of success in treatment of npAIR with rituximab monotherapy.^[5,8,9,10]^ However, recent results from a Phase-I/II clinical trial for npAIR patients treated with rituximab found that there was no definitive improvement noted in their series of five patients.^[11]^

Bortezomib is a proteasome inhibitor that promotes apoptosis of immortalized plasma cells in patients with multiple myeloma through increased expression of the tumor suppressor p53.^[12,13]^ Combination therapy with rituximab and bortezomib for a patient with non-paraneoplastic AIR has also been recently reported in a single case report; however, treatment did not result in significant clinical improvement.^[13]^

The purpose of this study was to show whether or not the combination of rituximab and bortezomib therapy has higher efficacy than rituximab monotherapy in the treatment of patients with non-paraneoplastic autoimmune retinopathy (npAIR).

##  METHODS

This was a single-center, retrospective observational case series of patients diagnosed with npAIR and treated between the period of January 2014 and October 2019. Approval of this study for chart review was obtained through the New England Institutional Review Board and performed in accordance with the Declaration of Helsinki and was Health Insurance Portability and Accountability Act compliant.

We performed a chart review for all cases of npAIR. Four patients (eight eyes) were selected based on the following inclusion criteria: (1) patients who had been treated with rituximab and bortezomib combination therapy or rituximab monotherapy with no other concomitant immunomodulatory therapy agents; (2) patients who had at least one year follow-up period for the aforementioned IMT regimens; (3) patients fulfilling the essential diagnostic criteria suggested for the diagnosis of AIR,^[6]^ namely: ERG abnormality with or without visual field abnormality, presence of serum antiretinal antibodies, absence of fundus lesions or retinal pathology that could explain the visual symptoms, absence of overt intraocular inflammation, and absence of systemic malignancy.

All patients were screened for malignancy including, but not limited to, computerized tomography scans of the head & neck, chest, abdomen, and pelvis. No patient had a prior history of cancer or melanoma. Supportive criteria^[6]^ were acute (0-3 months) or sub-acute (3-6 months) symptoms of one of the following: photopsias, scotomas, dyschromatopsia, or nyctalopia, and presence of personal or family history of systemic autoimmune disease. Core diagnostic tests included: optical coherence tomography (OCT) of macula, fundus autofluorescence (FAF), fluorescein angiography (FA), and indocyanine green angiography (ICGA).^[6]^ Patient demographics including treatment regimen prior to initiation of treatment, duration of each regimen, and side effects profile, all of which were noted for each patient during the follow-up period. A complete work-up for infectious and noninfectious uveitis had been done at the primary visit. OCT of macula, FAF, FA, and ICGA had also been performed to rule out occult inflammation in the retinal vessels, retina, and choroid. Blood samples were also sent to the Ocular Immunology Laboratory at Oregon Health Sciences University (Portland, Oregon) for detection of ARAs via Western Blot Analysis.^[14]^ Antiretinal and anti-optic nerve antibody bands were recorded based on their molecular weights (kDa) at the initial and final visits, where available.

The best-corrected visual acuity (BCVA) and related logarithm of the minimum angle of resolution (LogMAR), Humphrey visual field (Carl Zeiss Meditec, Inc., Dublin, CA, USA) mean deviation (MD) and pattern standard deviation (PSD), and full-field electroretinography (SG-2002, LKC Technologies) parameters including dim scotopic b-wave amplitude, bright scotopic a-wave and b-wave amplitudes, 30-Hz flicker amplitude, and 30-Hz flicker implicit time were recorded. Single flash photopic b-wave amplitude was only considered when 30-Hz flicker parameters were moving in opposite directions.^[15]^

The treatment protocol for rituximab (RituxanⓇ, Genentech, South San Francisco, CA, USA) was 375mg/m^2^ every week for eight weeks followed by 375mg/m^2^ monthly, a protocol that is frequently used to treat ocular cicatricial pemphigoid.^[16]^ The treatment protocol for bortezomib (VelcadeⓇ, Takeda) was 1.3 mg/m^2^ subcutaneously (SC) weekly for four weeks followed by one week off, a modified maintenance dosing protocol for multiple myeloma.^[17]^ This cycle was repeated following the one week off. Patients were monitored for occult side effects at each visit with adverse effects questioning, complete blood count, blood urea nitrogen, creatinine, and liver function tests.

For uniformity of assessment of findings, we described patient outcomes as either improved, stable, or progressive based on a combination of parameters: BCVA, HVF parameters, and ERG findings. *Improvement* was defined as (1) improvement of BCVA by at least two lines from baseline, (2) a decreased density or localization of scotomas on visual field perimetry or improvement in the mean deviation (MD) by ≥ 3dB, or (3) improved ERG parameters of ≥ 25% at last visit compared to baseline. *Stability* was defined as (1) BCVA within one line from baseline, (2) no change of density or localization of scotomas and/or MD within 3dB from baseline, or (3) ERG parameters change within 25% at last visit compared to baseline ERG. The criteria for test–retest reliability coefficients used for HVF and ERG in the study was based on prior publication in patients with glaucoma and retinitis pigmentosa, respectively.^[18,19]^ *Progression* was defined as patients who did not meet the criteria for either stability or improvement.

We also selected two historical patients from our previous study who were diagnosed with npAIR based on a consensus of clinicians and researchers^[6]^ and that had been treated with rituximab monotherapy.^[7]^ Since other patients in that study had been diagnosed with uveitis and retinal vasculitis, and their npAIR was secondary to ocular inflammatory diseases contrary to the consensus of clinicians and researchers,^[6]^ we were able to include only these two patients in this study.

The higher efficacy of combination therapy in comparison to monotherapy for each visual function parameter was defined as stability/improvement of that parameter in at least two or more eyes (33.3%). We decided to continue with stability/improvement of two or more eyes as the measurement to determine higher efficacy based on Simon's two-stage design which involves testing of a null hypothesis.^[20]^

##  RESULTS

### Case 1

**Figure 1:**
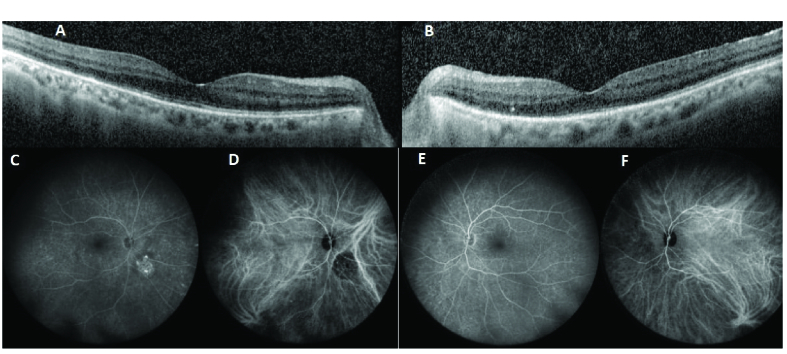
Macular optical coherence tomography (OCT) of right (A) and left (B) eyes, fundus fluorescein angiography (FFA) of the right (C) and left (E) eyes, and indocyanine green angiography (ICGA) of the right (D) and left ((F) eyes. They are all within the normal limit except for an atrophic unrelated lesion inferonasal to the optic nerve head in the right eye.

**Figure 2:**
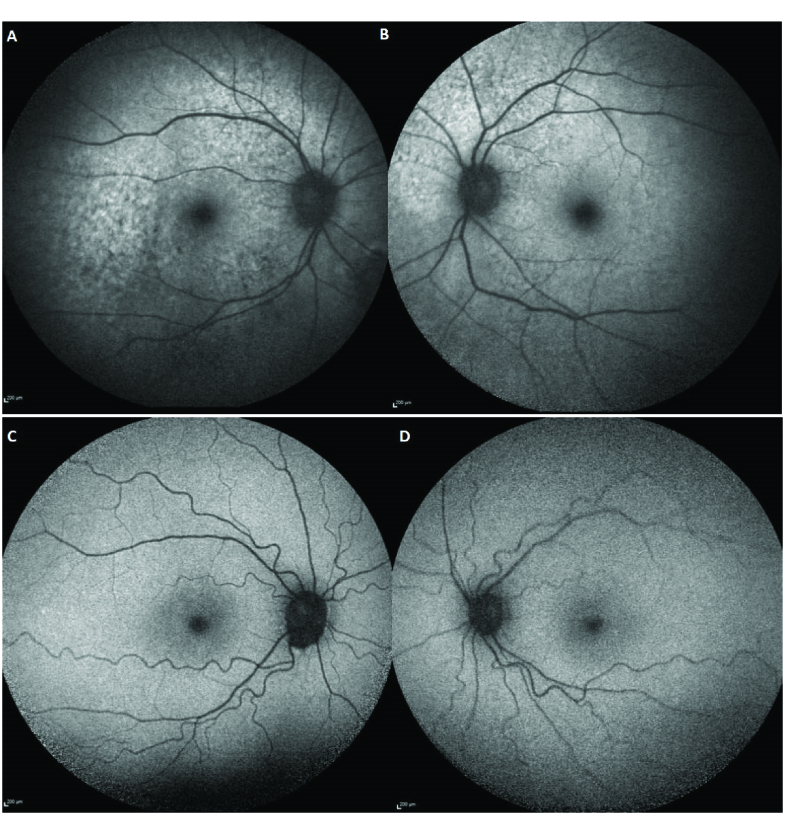
Blue autofluorence (BAF) of the right (A) and left (B) eyes of patient 1 which demonstrates hypo-autofluorescence around the arcades, around the optic nerve, and in macula of both eyes. BAF of the right (C) and Left (D) eyes of patient 2 does not show any changes in both eyes.

**Figure 3:**
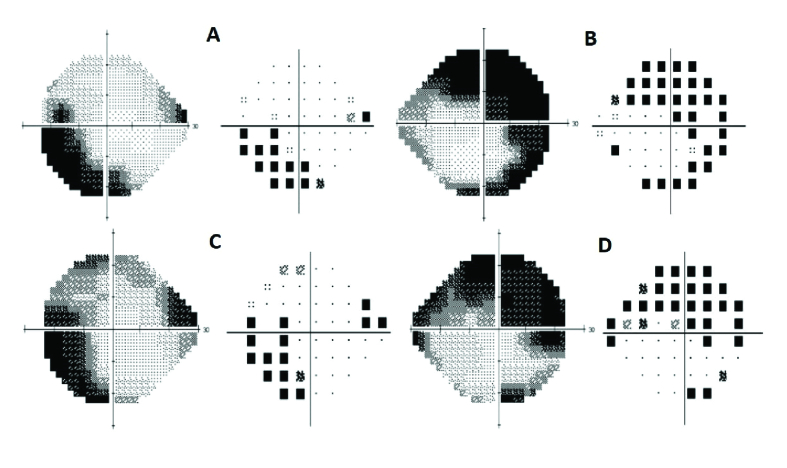
Humphrey visual field 24-II (pattern deviation plot) of the left (A) and right (B) eyes before treatment and the left (C) and right (D) eyes after treatment. They show stability of the visual field during the course of treatment.

A 72-year-old female with a four-year history of blurry vision OS (left eye) and flashes of light OD (right eye) was referred to us for evaluation. She was previously seen by a retinal specialist who suspected AIR and thus tested for anti-retinal antibodies through Oregon Labs, yielding positive findings. She was started on oral prednisone at 40 mg daily with a taper; however, symptoms persisted, thus prompting referral. An extensive evaluation for occult malignancy was done and results were negative. The patient had a past medical history of polymyalgia rheumatica and hypertension, with a pertinent past ocular history of choroidal nevus OD, stable since age 18. Figure 1shows FA, macular OCT, and ICG of both eyes at the initial visit. FA and ICG did not show any abnormalities indicating other diagnoses, and macular OCT did not demonstrate outer retinal changes which can happen in npAIR. Blue autofluorescence (BAF) demonstrated hypo-autofluorescence around the arcades, around the optic nerve, and in the macula of both eyes as signs of retinal pigment epithelium changes secondary to outer retinal disruption and changes^[21]^ [Figure 2A & 2B] Treatment with monthly rituximab 600 mg IV and bortezomib 2 mg SC was initiated. Six months after starting rituximab and bortezomib, the patient noticed improvement in flashes of light. Table 1shows BCVA, HVF MD and PSD, 30-Hz flicker amplitude, and implicit time at presentation, six months and twelve months after starting the treatment. She is currently under the same therapy. Before starting the treatment, she was positive for enolase and pyruvate kinase; however, at the last follow-up visit, she was positive for only enolase [Table 2]. Considering subjective improvement and stability of BCVA OU, HVF MD OD, and 30-Hz flicker implicit time OU (both eyes) at one year, this patient responded to rituximab and bortezomib combination therapy. Figure 3 demonstrates HVF at the initial and 12-month follow-up visits. No side effects were reported.

### Case 2 

**Figure 4:**
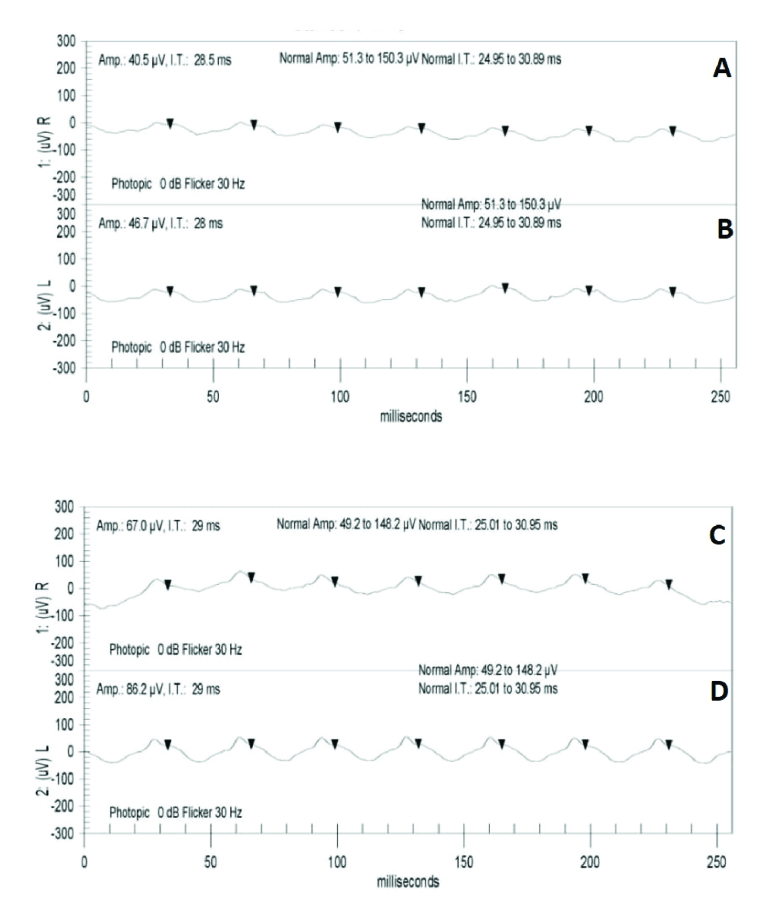
30-Hz flicker electroretinography (ERG) of the right (A) and left (B) eyes before starting treatment and the right (C) and left (D) eyes 12 months after treatment. They show improvement in 30-Hz flicker amplitude and stability in 30-Hz flicker implicit time in both eyes.

**Figure 5:**
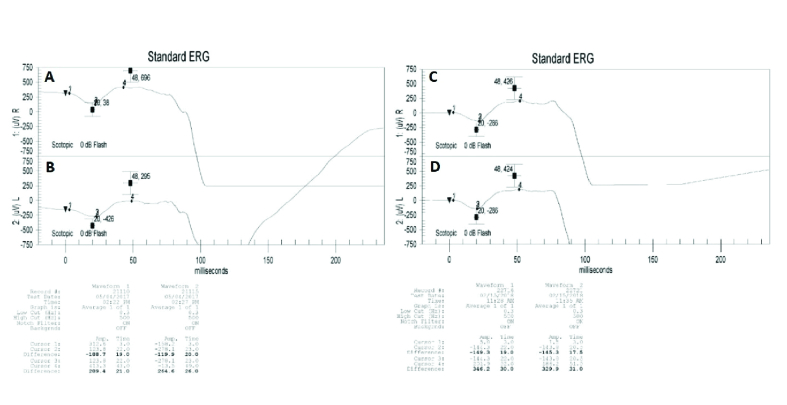
Scotopic combined cone and rod electroretinography (ERG) of the right (A) and left (B) eyes before starting treatment and the right (C) and left (D) eyes 12 months after treatment, which shows an improvement in b-wave amplitude out of proportion in comparison to a-wave amplitude in both eyes.

A 44-year-old male was seen at our center with a nine-month history of dimming vision, described as “rooms appear darker than before,” following a complicated ethmoid sinus infection occurring a few weeks after receiving the flu vaccine. Prior to consulting, the patient had been seen by numerous ophthalmologists and all test results were found to be normal. Testing included OCT Macula and Fluorescein Angiography. BAF did not depict pigmentary changes or hypo-autofluorescence around the arcades and the optic nerve, or in the macula ^[21]^ [Figure 2C &2D]. Brain and orbital Magnetic Resonance Imaging (MRI) were also done with normal results. Table 1shows BCVA, HVF MD and PSD, scotopic b-wave amplitude, scotopic combined rod and cone response a-wave and b-wave amplitudes, 30-Hz flicker amplitude and implicit time at presentation. He was diagnosed with AIR and a trial with oral prednisone at 60 mg/day was started. Investigation to rule out underlying malignancy was performed with no malignancy found. Combination of rituximab and bortezomib therapy was then initiated. Six months after the initiation of therapy, repeat HVF SITA-SWAP was performed that showed stable findings compared to initial testing [Table 1]. The patient is currently under the same therapy. He was positive for 23 kDa both before starting the treatment and at the last follow-up visit [Table 2]. Figure 4shows improvement in 30-Hz flicker amplitude with stable implicit time from the first visit to 12 months' follow-up visit in both eyes. Figure 5 demonstrates improvement in scotopic combined rod and cone b-wave amplitude out of proportion compared to the improvement in a-wave amplitude. Based on subjective improvement and stability/improvement in all visual function parameters, excluding scotopic b-wave amplitude OU, this patient was successfully treated with rituximab and bortezomib combination therapy. No side effects were reported.

### Case 3

A 17-year-old male was referred to us for co-management. He presented with a one-year history of peripheral visual field loss associated with intermittent eye pain; however, he denied nyctalopia and hemeralopia. He was subsequently seen by a retina specialist who was suspicious of AIR, with noted findings of extinguished ERG. Investigations to detect an occult malignancy were then carried out by his primary physician, which were normal. He was diagnosed with npAIR and then started on oral prednisone at 60 mg/day. Testing was repeated after one month with noted improvement in ERG and HVF. He came back four months later with new onset floaters and increased peripheral vision loss OS > OD, which was noted to occur upon discontinuation of prednisone. His local ophthalmologist then restarted oral prednisone at 60 mg/day. Oral prednisone was continued with a plan to start rituximab and bortezomib as immunosuppressive therapy. Table 1 shows BCVA, HVF MD and PSD, scotopic b-wave amplitude, scotopic combined rod and cone response a-wave and b-wave amplitudes, 30-Hz flicker amplitude and implicit time at presentation. Six months after the initiation of therapy, repeat HVF SITA-SWAP was done which showed stable findings compared to initial testing. 30-Hertz flicker ERG revealed good implicit times OU with some decrease in amplitude OD [Table 1]. Before starting the treatment, the patient was positive for 21–22 and 72 kDs; however, at the last follow-up visit, all antibodies had disappeared [Table 2]. This patient was considered treatment failure for combined rituximab and bortezomib therapy since his subjective symptoms, including peripheral vision loss, progressive night vision blindness, and new light flashes OU, and HVF MD OU got worse despite stability/improvement in BCVA and all of ERG parameters except 30 Hz amplitude OD at the 12-month follow-up visit. No side effects were reported.

### Case 4

A 59-year-old woman was referred to us for evaluation of progressive blurry vision OU over about a three-month period.She had been recently diagnosed with systemic lupus erythematosus. MRI did not reveal a central nervous system (CNS) cause for her condition. Based on her diagnosis, she was started on mycophenolate mofetil. The patient continued to experience significant worsening vision in her right eye despite maximum dose of mycophenolate mofetil therapy for about 12 weeks. Given progressive vision loss with no clinical findings, past medical history of systemic lupus erythematosus, and ancillary testing parameters, antiretinal antibody was checked and came back positive [Table 2]. The patient was started on rituximab monotherapy which was given weekly for 6 weeks and then monthly for the following 18 months. Table 1demonstrates BCVA, HVF, MD and PSD, scotopic b-wave amplitude, scotopic combined rod and cone response a-wave and b-wave amplitudes, 30-Hz flicker amplitude and implicit time at the initial, sixth, and twelfth-month follow-up visits. Before starting the treatment, the patient was positive for enolase, which remained positive throughout the study period. Based on subjective improvement and stability of BCVA OU, HVF MD OU, b-scotopic amplitude OU, and 30-Hz flicker implicit time OU, this case was successfully treated with rituximab therapy. No side effects were reported.

### Historical cases ${}^{\left[7\right]}$

We also included two historical cases who had been treated with rituximab monotherapy in this study.^[7]^

### Historical case 1 ${}^{\left[7\right]}$

A 51-year-old woman was diagnosed with npAIR and started on rituximab monotherapy. She was treated with rituximab monotherapy for 20 months. During the study period, BCVA was stable in both eyes, HVF MD was stable OS and got worse OD, and HVF PSD was stable in both eyes. Scotopic b-wave amplitude was stable OD and got worse OS. Scotopic combined a-wave and b-wave amplitudes were stable in both eyes, except for the right eye a-wave amplitude which got worse. 30-Hz flicker amplitude and implicit time improved OD, but amplitude and implicit time OS got worse and was stable, respectively. ARA bands were positive for aldolase and enolase before starting treatment; however, at the last follow-up visit, the patient was positive for enolase. More detail was not available about this patient. This patient was considered stable based on the definition of stability.^[7]^

**Table:** Visual function parameters at baseline, 6-month, and 12-month visits in patients on rituximab and bortezomib combination therapy (patient 1–3) and rituximab monotherapy (patient 4).

	**BCVA**		**HVF**				**ERG**									
**Baseline visit**	**OD**	**OS**	**MD OD**	**MD OS**	**PSD OD**	**PSD OS**	**SCO B OD**	**SCO B OS**	**COM A OD**	**COM A OS**	**COM B OD**	**COM B OS**	**30-Hz AMP OD**	**30-Hz AMP OS**	**30-Hz IMP OD**	**30-Hz IMP OS**
**Patient 1**	0.2	0.1	–4.38	–3.42	1.95	3.84	N/A	N/A	N/A	N/A	N/A	N/A	9.37	10.16	27.58	28.76
**Patient 2**	–0.1	0	–0.62	–0.11	2.9	2.54	131.5	99.8	122.1	121.3	284.9	242.8	45.5	46.7	28.5	28
**Patient 3**	0	0	–8.25	–9.05	6.45	9.89	8.2	14.1	–9.9	–16.2	196.6	172.2	39.19	24.86	32.5	32.5
**Patient 4**	0	0.2	–6.41	–20.1	8.39	12.35	89.6	43.9	124.8	101.7	174.6	152	52.8	44.1	30.5	30.5
**6-month visit**	**OD**	**OS**	**MD OD**	**MD OS**	**PSD OD**	**PSD OS**	**SCO B OD**	**SCO B OS**	**COM A OD**	**COM A OS**	**COM B OD**	**COM B OS**	**30-Hz AMP OD**	**30-Hz AMP OS**	**30-Hz IMP OD**	**30-Hz IMP OS**
**Patient 1**	0.4	0.3	–5.6	–3.17	2.3	1.64	N/A	N/A	N/A	N/A	N/A	N/A	4.63	5.16	275.0	24.1
**Patient 2**	–0.1	–0.1	–0.46	–0.26	2.24	2.29	194.5	99.4	–188	–119.9	289.4	264.6	45.9	38.3	30	29.5
**Patient 3**	0	0	–6.12	–15.88	8.58	16.6	130.9	135.	185.	–141.1	421.1	335.3	29.7	26.92	26.5	29
**Patient 4**	0	0	–8.29	–19.86	8.3	13.2	207.8	69.6	106.	–78	151.2	126.4	26.7	21.6	32.5	31
**12-month visit**	**OD**	**OS**	**MD OD**	**MD OS**	**PSD OD**	**PSD OS**	**SCO B OD**	**SCO B OS**	**COM A OD**	**COM A OS**	**COM B OD**	**COM B OS**	**30-Hz AMP OD**	**30-Hz AMP OS**	**30-Hz IMP OD**	**30-Hz IMP OS**
**Patient 1**	0.1	0.1	–4.4	–8.67	3.56	7.31	N/A	N/A	N/A	N/A	N/A	N/A	6.08	5.84	287.4	290.15
**Patient 2**	0	–0.1	1.92	–0.98	2.91	2.08	58.1	69.4	149.3	145.3	346.2	329.9	64.2	75.9	28.5	27.5
**Patient 3**	0	0.1	–6.1	–15.8	8.58	16.64	81.4	120.	–67.8	137.6	305.6	328.7	16.4	25.7	28	29
**Patient 4**	0	0.1	–6.9	–20	7.43	12.41	69.3	54.3	88.50	0	118.8	83.5	39.9	26.7	31.5	31.5
A, A-wave; AMP, amplitude; B, B-wave; BCVA, best-corrected visual acuity; COM, combined; ERG, electroretinography; HVF, Humphrey visual filed; IMP, implicit time; OD, right eye; OS, left eye; SCO, scotopic																

**Table 2:** Changes in antiretinal and anti-optic nerve antibodies from baseline to 6-months to 12-months in patients on rituximab and bortezomib combination therapy (patient 1–3) and rituximab monotherapy (patient 4).


	**At baseline**	**6-month follow-up**	**12-month follow-up**
**Patient 1**	Retina enolase, Pyruvate kinase; ON negative	Positive enolase; Negative ON	Positive enolase; ON 82 Kda
**Patient 2**	Anti-retina: Positive 23 kDa and anti-ON 20 kDa, 23 kDa, 32 kDa, 37 kDa	Negative ON and retina	Positive retina 23 kDa, no reoverein; ON 35 kDa and 37 kDa
**Patient 3**	Retina: 21-22 kDa and 72 kDa	Retina 30 kDa (CAII), 36 kDa (GAPDH, 45 kDa (arrestin) and 72 kDa	N/A
**Patient 4**	46 kDa (enolase); ON: 36 Kda, 46 kDa, 50 kDa, 62 kDa	Enolase; Negative ON	Retina 46 kDa (enolase); ON 30 kDa, 36 kDa, 46 kDa, 55 kDa
**Patient 1 (H)**	40 kDa (aldolase) and 46 kDa (enolase)	N/A	46 kDa (enolase)
**Patient 2 (H)**	33-35 kDa (carbonic anhydrase)	N/A	None
	
	

**Table 3:** Changes in visual function parameters from baseline to twelve-month follow-up visit.


	**BCVA**		**HVF**				**ERG**									
	**OD**	**OS**	**MD OD**	**MD OS**	**SCO B OD**	**SCO B OS**	**COM A OD**	**COM A OS**	**COM B OD**	**COM B OS**	**30-Hz AMP OD**	**30-Hz AMP OS**	**30-Hz IMP OD**	**30-Hz IMP OS**
**Patient 1**	STA	STA	STA	DEC	–	–	–	–	–	–	DEC	DEC	STA	STA
**Patient 2**	STA	STA	STA	STA	DEC	DEC	IMP	IMP	IMP	IMP	IMP	IMP	STA	STA
**Patient 3**	DEC	DEC	DEC	DEC	IMP	IMP	IMP	IMP	IMP	IMP	DEC	STA	STA	STA
**Patient 4**	STA	STA	STA	STA	STA	STA	DEC	STA	DEC	DEC	DEC	DEC	STA	STA
**Patient 5**	STA	STA	STA	STA	STA	DEC	DEC	STA	DEC	STA	IMP	DEC	IMP	STA
**Patient 6**	STA	STA	STA	STA	DEC	STA	DEC	DEC	DEC	DEC	STA	DEC	STA	STA
A, A-wave; AMP, amplitude; B, B-wave; BCVA, best-corrected visual acuity; COM, combined; DEC, decreased; ERG, electroretinography; HVF, Humphrey visual filed; IMP, implicit time; IMP, improved; OD, right eye; OS, left eye; SCO, scotopic; STA, stable																

**Table 4:** Response of visual function parameters to rituximab and bortezomib combination therapy versus rituximab monotherapy.


	**Rituximab monotherapy**	**Rituximab + Borzomib**	**Superiority of treatment**
**BCVA**	6/6	6/6	R = R + B
**HVF MD**	4/6	5/6	R = R + b
**HVF PSD**	5/6	5/6	R = R + B
**Scotopic B-wave**	2/4	4/6	**R > R + b**
**Combined A-wave**	4/4	2/6	**R < R + B**
**Combined B-wave**	4/4	2/6	**R < R + B**
**30-Hz Amplitude**	3/6	2/6	R = R + B
**30-Hz Implicit time**	6/6	6/6	R = R + B
BCVA, best-corrected visual acuity; B, bortezomib; HVF, Humphrey visual field; MD, mean deviation; Pattern standard deviation; R, rituximab			

### Historical case 2 ${}^{\left[7\right]}$

A 73-year-old woman was diagnosed with npAIR and was started on rituximab therapy. She was treated with rituximab monotherapy for 11 months; however, the treatment was stopped due to insurance problem. During the study period, BCVA got worse OD and was stable OS. HVF MD and PSD were stable in both eyes. Scotopic b-wave amplitude, scotopic combined a-wave and b-wave amplitudes got worse in both eyes. 30-Hz flicker amplitude and implicit time was stable OD, but amplitude and implicit time OS got worse and improved, respectively. ARA bands were positive for carbonic anhydrase before starting treatment, which became negative at six months. More detail was not available about this patient. This patient was considered stable based on the definition of stability.^[7]^

We calculated the number of eyes in each group (rituximab and bortezomib combination therapy versus rituximab monotherapy) for all studied parameters that improved or were stable during the one-year follow-up and these are demonstrated in Table 3. Table 4 demonstrates the response of visual function parameters to rituximab and bortezomib combination therapy versus rituximab monotherapy.

##  DISCUSSION

Autoimmune retinopathy (AIR) is a rare disease of the retina that is potentially blinding. Despite having been first defined over 20 years ago, there is still no consensus between ophthalmologists and ocular immunologists on how to correctly diagnose and treat this condition.

There are a few case reports and case series that studied the efficacy and safety of rituximab in npAIR.^[5,6,7,8,11]^ Our knowledge about the combination of rituximab and bortezomib is even more limited.^[7,13]^ We previously reported this combination therapy for patients with secondary npAIR and underlying uveitis (birdshot chorioretinopathy).^[7]^

Since there were few patients on each treatment regimen, we compared the number of eyes which were stable/improved for each visual function parameter between the two groups. We did not find any difference in most of the studied parameters including BCVA, MD in HVF, 30-Hz flicker amplitude and implicit time, as well as scotopic b-wave amplitude; thus, the combination therapy was not found to be more effective than rituximab monotherapy for these parameters. Comparatively, the scotopic combined rod and cone response a-wave and b-wave amplitudes were improved in the combination regimen when they were available (patient 2 and patient 3) and showed no improvement in eyes in rituximab monotherapy. The average improvement in b-wave amplitude (50.7% ± 29.4% [range, 25–90%]) was higher compared to the average improvement in the a-wave amplitude (35.7% ± 9.74% [range, 25–63%]). For rituximab monotherapy, it was stable in only two out of the six eyes. If we consider the average improvement of 50.7% in b-wave and 25% in wave in combination therapy group, we may conclude that when the inner retina or bipolar cells are involved, combination therapy may lead to more significant results. This is a hypothesis at this point due to small sample size and an overlap between the a-wave and b-wave ranges. The anti-retinal antibodies target antigens specific to the inner retina and bipolar cells, which can provide a good explanation for this difference; however, this finding should be examined with more potent studies and larger population since only one eye of a historical patient and none of the eyes of the studied patients showed negative b-wave on scotopic combined rod and cone response, possibly contradicting our finding.

Benson and colleagues reported an npAIR patient treated with rituximab (1000 mg IV on days 1 and 15) in combination with bortezomib (1.5 mg/m ${}^{2}$ on days 1, 8, and 15 out of a 28-day cycle for a total of 6 cycles).^[13]^ They concluded that combined rituximab and bortezomib treatment did not result in significant clinical improvement as there was evidence of disease progression. However, there are some flaws in this study which make their results less reliable. First, there was extensive leakage in the periphery of the retina in both eyes and impressive leakage from the right eye optic nerve head, neither of which resolved after the combination therapy. These findings make the diagnosis of npAIR less likely due to the previously described consensus. Furthermore, they did not consider test–retest reliability in BCVA, ERG, and HVF. In contrast to their results, our study showed improvements in three or more ERG parameters for two out of three patients. We were also able to retain our patients' vision at normal or near normal range in the combination group. Similarly, HVF parameters were also stable in all but two eyes.

We also compared our results with the largest npAIR study conducted by Davoodi et al, who found stability of ERG and MD in their patients;^[9]^ however, Davoodi and colleagues also included paraneoplastic AIR patients in their study. They employed rituximab in combination with mycophenolate mofetil, methotrexate, cyclophosphamide, bortezomib, and intravenous immunoglobulin (IVIg) in their patients. The main parameter focused on in that study was the visual acuity; however, this is not a good parameter to focus on as some npAIR patients, including all patients in the Davoodi et al case series, complain about other visual function parameters including peripheral and night visions, despite having normal central vision. The threshold for stability in their study also differed from ours for ERG, being at 40% instead of 25%. This consequently shows that the results are more favorable since the desired result of treatment in npAIR patients is stability or improvement of visual function parameters; however, with a higher threshold for stability (40% vs 25%), the number of eyes with progression will become less. Based on this discrepancy, we were not able to compare the ERG in our cohort with the Davoodi et al study. Davoodi and colleagues used the same criteria for visual field progression as our study. No eyes showed improvement in visual field during the course of treatment. Our results in both the combination therapy and rituximab monotherapy were similar to their results; they employed other conventional IMTs, biologic response modifier agents, and cytotoxic agents. Based on other agents they employed including cytotoxic agents, it might be concluded that our monthly rituximab infusion protocol as monotherapy or in combination with bortezomib is more effective than the two different rituximab therapy protocols that they employed in their study since none of their patients showed significant changes ( > 40%) in any ERG amplitudes or implicit times over the course of rituximab treatment.

It is important to note that antiretinal and anti-optic nerve antibody bands can be present in normal population and their presence does not specifically indicate AIR.^[22]^ Similar to the Boudreault et al study,^[5]^ we did not find any correlation between changes in the types and levels of antibodies. Boudrealt and colleagues explained this by mentioning that CD20 receptor may not be present on antibody-producing plasma cells, and that the level of antibodies are not expected to decrease.^[5]^ However, our study patients in the combination therapy group may be contradictory to their explanation for the lack of antiretinal antibody clearing, since bortezomib is a proteosome inhibitor which prevents the degradation of pro-apaptotic factors and triggers programmed cell death of plasma cells. This contradicts Bourdrealt et al's hypothesis about antibody production by CD20 negative plasma cells as a cause for rituximab failure.

Inner retinal layers can be involed more commonly in MAR^[8]^ and less commonly in npAIR.^[1]^ Changes in scotopic combined rod and cone response b-wave amplitude are characteristic of the inner retina. Interestingly, scotopic combined rod and cone response b-wave amplitude was available in two patients and four eyes for the combination therapy, all of which showed improvement. For patients in the monotherapy group, scotopic combined rod and cone response b-wave amplitudes was stable in one eye of each patient and worsened in the other eyes. A decrease in scotopic combined rod and cone response b-wave amplitudes might indicate a need for combination therapy; however, more potent studies should be done to prove this hypothesis. Additionally, npAIR with inner retina involvement might be more similar to MAR (faster progression) in comparison to other types of npAIR.

We might be criticized for putting more value on ERG and HFV for diagnosis of npAIR; however, we followed the diagnostic criteria of npAIR based on Fox et al's^[6]^ study in which changes in ERG parameters with or without changes in HVF parameters is a major criterion, and FA, OCT macula, FAF are core diagnostic tests. This is reasonable since npAIR is a generalized dysfunction of retina and ERG is a mass response which is not generally affected by local diseases of retina. Given the consensus on the diagnosis and management of npAIR study,^[6]^ patients with normal ERG do not fulfil the criteria for diagnosis of npAIR. We recently published a case report which may help in understanding the aforementioned explanation.^[23]^

There were a number of limitations to this study. The retrospective nature is its most important drawback. The limited number of patients and use of historical cases was another important limitation; however, considering all these limitations, a study of six patients with rare orphan disease and exclusive treatment with rituximab monotherapy or rituximab and bortezomib combination therapy was a strong point and made this study different from all past case series. We also admit that we weighted ERG and HVF over FA, FAF, ICG, and macular OCT since we were following the essential diagnostic criteria suggested for the diagnosis of AIR.^[6]^

In conclusion, the combination of rituximab and bortezomib therapy might not be more effective than rituximab monotherapy for most of the visual function parameters in npAIR patients; however, this combination therapy might be more effective than rituximab monotherapy when combined scotopic rod and cone-dependent a-wave and b-wave amplitudes are reduced.

##  Ethical Considerations

This study was approved by the New England Institutional Review Board, which issued a waiver of informed consent for the retrospective chart review analysis. This study was performed in accordance with the Helsinki Declaration of 1964, and its later amendments. All participants provided consent for publication if any identifying information is included in the manuscript.

##  Financial Support and Sponsorship

None.

##  Conflicts of Interest

None of the authors have any conflict of interest with the content of this manuscript.

##  Disclosures

Dr. C Stephen Foster declares the following:

Consultancies with Aldeyra Therapeutics (Lexington, MA), Allakos (Redwood City, CA), Bausch & Lomb Surgical, Inc. (Rancho Cucamonga, CA), Eyegate Pharma (Waltham, MA), Genentech (South San Francisco, CA), Novartis (Cambridge, MA), pSivida (Watertown, MA)

Grants or grants pending with Aciont (Salt Lake City, UT), Alcon (Aliso Viejo, CA), Aldeyra Therapeutics (Lexington, MA), Bausch & Lomb (Rochester, NY), Clearside Biomedical (Alpharetta, GA), Dompé pharmaceutical (Milan, Italy), Eyegate Pharma (Waltham, MA), Mallinckrodt pharmaceuticals (Staines-upon-Thames, UK), Novartis Pharmaceuticals (Cambridge, MA), pSivida (Watertown, MA), Santen (Osaka, Japan).

Payment for lectures including service on speaking bureaus: Alcon (Aliso Viejo, CA), Allergan (Dublin, Ireland), Mallinckrodt pharmaceuticals (Staines-upon-Thames, UK).

Stock or Stock Options: Eyegate Pharma (Waltham, MA)

Dr. Stephen D. Anesi declares the following:

Consultancies with Santen (Osaka, Japan), Mallinckrodt (Staines-upon-Thames, UK), Allakos (Redwood City, CA), Eyepoint (Watertown, MA), and Takeda (Tokyo, Japan).

Speakerships with AbbVie (Chicago, IL), Mallinckrodt (Staines-upon-Thames, UK), and Eyepoint (Watertown, MA).

Dr. Peter Chang declares the following:

Consultancies with Eyepoint (Watertown, MA) and Alimera (Alpharetta, GA). Speakerships with AbbVie (Chicago, IL), Mallinckrodt (Staines-upon-Thames, UK), and Eyepoint (Watertown, MA).

## References

[B1] Mizener JB, Kimura AE, Adamus G, Thirkill CE, Goeken JA, Kardon RH (1997). Autoimmune retinopathy in the absence of cancer. Am J Ophthalmol.

[B2] Takiuti JT, Takahashi VK, Xu CL, Jauregui R, Tsang SH (2019). Non-paraneoplastic related retinopathy: Clinical challenges and review. Ophthalmic Genet.

[B3] Grange L, Dalal M, Nussenblatt RB, Sen HN (2014). Autoimmune retinopathy. Am J Ophthalmol.

[B4] Sobrin L

[B5] Boudreault K, Justus S, Sengillo JD, Schuerch K, Lee W, Cabral T, et al (2017). Efficacy of rituximab in non-paraneoplastic autoimmune retinopathy. Orphanet J Rare Dis.

[B6] Fox AR, Gordon LK, Heckenlively JR, Davis JL, Goldstein DA, Lowder CY, et al (2016). Consensus on the diagnosis and management of nonparaneoplastic autoimmune retinopathy using a modified delphi approach. Am J Ophthalmol.

[B7] Maleki A, Lamba N, Ma L, Lee S, Schmidt A, Foster CS (2017). Rituximab as a monotherapy or in combination therapy for the treatment of non-paraneoplastic autoimmune retinopathy. Clin Opthalmol.

[B8] Fox A, Jeffrey B, Hasni S, Nussenblatt R, Sen HN (2015). Rituximab treatment for nonparaneoplastic autoimmune retinopathy. Can J Ophthalmol.

[B9] Davoudi S, Ebrahimiadib N, Yasa C, Sevgi DD, Roohipoor R, Papavasilieou E, et al (2017). Outcomes in autoimmune retinopathy patients treated with Rituximab. Am J Ophthalmol.

[B10] Uludag G, Onal S, Arf S, Sayman Muslubas I, Selcukbiricik F, Koc Akbay A, et al (2016). Electroretinographic improvement after rituximab therapy in a patient with autoimmune retinopathy. Am J Ophthalmol Case Rep.

[B11] Armbrust KR, Fox AR, Jeffrey BG, Sherry P, Sen HN (2020). Rituximab for autoimmune retinopathy: Results of a Phase I/II clinical trial. Taiwan J Ophthalmol.

[B12] Russo A, Fratto ME, Bazan V, Schiró V, Agnese V, Cicero G, et al (2007). Targeting apoptosis in solid tumors: The role of bortezomib from preclinical to clinical evidence. Expert Opin Ther Targets.

[B13] Benson MD, Plemel DJ, Yacyshyn E, Sandhu I, MacDonald IM, Baker CF (2020). Combination treatment with Rituximab and Bortezomib in a patient with non-paraneoplastic autoimmune retinopathy. Ocul Immunol Inflamm.

[B14] Adamus G, Ren G, Weleber RG (2004). Autoantibodies against retinal proteins in paraneoplastic and autoimmune retinopathy. BMC Ophthalmol.

[B15] McCulloch DL, Marmor MF, Brigell MG, Hamilton R, Holder GE, Tzekov R, et al (2015). ISCEV Standard for full-field clinical electroretinography (2015 update). Doc Ophthalmol.

[B16] Cao JH, Oray M, Cocho L, Foster CS (2016). Rituximab in the treatment of refractory noninfectious scleritis. Am J Ophthalmol.

[B17] Mohan M, Matin A, Davies FE (2017). Update on the optimal use of bortezomib in the treatment of multiple myeloma. Cancer Manag Res.

[B18] Fishman GA, Chappelow AV, Anderson RJ, Rotenstreich Y, Derlacki DJ (2005). Short-term inter-visit variability of erg amplitudes in normal subjects and patients with retinitis pigmentosa. Retina.

[B19] Mills RP, Budenz DL, Lee PP, Noecker RJ, Walt JG, Siegartel LR, et al (2006). Categorizing the stage of glaucoma from pre-diagnosis to end-stage disease. Am J Ophthalmol.

[B20] Simon R (1989). Optimal two-stage designs for phase II clinical trials. Control Clin Trials.

[B21] Khanna S, Martins A, Oakey Z, Mititelu M (2019). Non-paraneoplastic autoimmune retinopathy: Multimodal testing characteristics of 13 cases. J Ophthalmic Inflamm Infect.

[B22] Adamus G

[B23] Garcia CM, Maleki A, Look-Why S, Manhapra A, Durrani K, Foster CS

